# Comparison of Postoperative Quality of Life among Three Different Reconstruction Methods After Proximal Gastrectomy: Insights From the PGSAS Study

**DOI:** 10.1007/s00268-020-05629-5

**Published:** 2020-06-06

**Authors:** Hiroshi Yabusaki, Yasuhiro Kodera, Norimasa Fukushima, Naoki Hiki, Shinichi Kinami, Masashi Yoshida, Keishiro Aoyagi, Shuichi Ota, Hiroaki Hata, Hiroshi Noro, Atsushi Oshio, Koji Nakada

**Affiliations:** 1 Department of Gastroenterological Surgery, Niigata Cancer Center Hospital, 2-15-3, kawagishicho, chuoku, Niigata, 951-8566 Japan; 2 Department of Gastroenterological Surgery, Nagoya University Graduate School of Medicine, Nagoya, Japan; 3 Department of Surgery, Yamagata Prefectural Central Hospital, Yamagata, Japan; 4 Department of Upper Gastrointestinal Surgery, Kitasato University School of Medicine, Sagamihara, Kanagawa Japan; 5 Department of Surgical Oncology, Kanazawa Medical University, Kahoku, Ishikawa Japan; 6 Department of Surgery, International University of Health and Welfare Hospital, Otawara, Tochigi Japan; 7 Department of Surgery, Kurume University School of Medicine, Kurume, Japan; 8 Department of Surgery, Osaka Saiseikai - Noe Hospital, Osaka, Japan; 9 Department of Surgery, National Hospital Organization, Kyoto Medical Centre, Kyoto, Japan; 10 Department of Surgery, Japan Community Health Care Organization (JCHO), Osaka Hospital, Osaka, Japan; 11 Faculty of Letters, Arts and Sciences, Waseda University, Shinjuku, Tokyo Japan; 12 Department of Laboratory Medicine, Jikei University School of Medicine, Minato, Tokyo Japan

## Abstract

**Background:**

Proximal gastrectomy (PG) has become an increasingly preferred procedure for early cancer in the upper third of the stomach, owing to reportedly superior quality of life (QOL) after PG when compared with total gastrectomy. However, various methods of reconstruction have currently been proposed. We compared the postoperative QOL among the three different reconstruction methods after PG using the Postgastrectomy Syndrome Assessment Scale-45 (PGSAS-45) questionnaire.

**Methods:**

Post Gastrectomy Syndrome Assessment Study (PGSAS), a nationwide multi-institutional survey, was conducted to evaluate QOL using the PGSAS-45 among various types of gastrectomy. Of the 2,368 eligible data from the PGSAS survey, data from 193 patients who underwent PG were retrieved and used in the current study. The PGSAS-45 consists of 45 items including 22 original gastrectomy specific items in addition to the SF-8 and GSRS. These were consolidated into 19 main outcome measures pertaining postgastrectomy symptoms, amount of food ingested, quality of ingestion, work, and level of satisfaction for daily work, and the three reconstruction methods (*n* = 193; 115 esophago-gastrostomy [PGEG], 34 jejunal interposition [PGJI], and 44 jejunal pouch interposition [PGJPI]) were compared using PGSAS-45.

**Results:**

Size of the remnant stomach was significantly larger in PGEG, and significantly smaller in PGJI and PGJPI (*P* < 0.05). There was no difference in other patient background factors among the groups. EGJPI tended to be superior to PGEG in several of the 19 main outcome with marginal significance (*P* = 0.047–0.076).

**Conclusion:**

PGJPI appears to be the most favorable of the three reconstruction methods after PG especially when the size of remnant stomach is rather small.

**Trial registration number:**

UMIN-CTR #000002116 entitled as “A study to observe correlation between resection and reconstruction procedures employed for gastric neoplasms and development of postgastrectomy syndrome”

## Introduction

Although the relative frequency of early gastric cancer existing on one-thirds of upper part of the stomach has been increasing [1, 2], no standard surgical procedure has been proposed based on robust clinical data [3, 4]. Recently, Postgastrectomy Syndrome Working Party (PGSWP), a voluntary group of Japanese surgeons focused on relieving postgastrectomy symptoms, progressed Postgastrectomy Syndrome Assessment Scale-45 (PGSAS-45), a tool evaluating patient reported outcome among patients who underwent gastrectomy [5]. A comparison of retrospective data between total gastrectomy (TG) and proximal gastrectomy (PG) using PGSAS-45 revealed superiority of PG over TG regarding several primary outcomes [6].

PG was defined by the Japanese gastric cancer treatment guidelines version 4 [7] as a modified gastrectomy and was proposed as an option for cT1cN0 adenocarcinoma existing on one-thirds of upper part of the stomach provided over half of the distal stomach can be preserved. Considered as a function-preserving procedure, PG is now widely performed to improve postoperative quality of life (QOL). In truth, however, various reconstruction methods have been attempted following PG according to the preference of the surgeons, sometimes depending on factors such as the remnant stomach size. Reconstruction procedure ranges from esophago-gastrostomy (PGEG) [8, 9] usually with the anti-reflux methods (e.g., fundoplication or to create a His angle), to jejunal interposition method (PGJI) [10, 11], double tract method [12, 13], and jejunal pouch interposition method (PGJPI) [14, 15], of which the optimal method remains the matter of controversy.

The purpose of this study is to identify the most appropriate reconstruction method after PG using data from Postgastrectomy Syndrome Assessment Study (PGSAS) survey which is nationwide multi-institution surveillance of postgastrectomy patients in Japan using the PGSAS-45.

## Materials and methods

### Patients and eligibility criteria

Fifty-two institutions from all over Japan joined our surveillance. Questionnaire of the PGSAS-45 was delivered to 2,922 outpatients during from July 2009 to December 2010. Eligibility criteria were: (1) gastric cancer in stage IA or IB confirmed pathologically; (2) age from 20 to 75 years; (3) no experience of chemotherapy; (4) without recurrence or distant metastasis; (5) gastrectomy to be performed one year prior of the registration; (6) PS is 0 or 1 of ECOG; (7) sufficient ability to comprehend and answer to our forms; (8) without any medical record of other illnesses or previous surgical treatment that may affect their answers; (9) normal function of organs and mental state; and (10) supply of scripted informed consent. Patients with dual malignancy or concomitant resection of other organs (we permitted simultaneous resection equivalent for cholecystectomy) and we excepted those who underwent completion gastrectomy.

### Assessment of QOL

The PGSAS-45 that developed newly consisted of the SF-8; Short-Form Health Survey [16] and the GSRS; Gastrointestinal Symptom Rating Scale is a multi-dimensional QOL questionnaire [17]. The PGSAS-45 questionnaire includes 45 items, with 8 from the SF-8, 15 from the GSRS, and 22 original selected as clinically relevant by PGSWP (Table 1). The PGSAS-45 contains 23 items associated with postgastrectomy conditions (from 9 to 33), containing 15 from GSRS and 8 original.

**Table 1:** Structure of Postgastrectomy Syndrome Assessment Scale (PGSAS)-45

Domains	Items		Items		Subscales
QOL	SF-8 (QOL)	1	Physical functioning*	Five or six-point Likert scale	Physical component summary (PCS)* (item 1–8)
		2	Role physical*		Mental component summary (MCS)* (item 1–8)
		3	Bodily pain*		
		4	General health*		
		5	Vitality*		
		6	Social functioning*		
		7	Role emotional*		
		8	Mental health*		
Symptoms	GSRS Symptoms)	9	Abdominal pains	Seven-point Likert scale	Esophageal reflux subscale (item 10, 11, 13, 24)
		10	Heartburn	Except item 29 and 32	Abdominal pain subscale (item 9, 12, 28)
		11	Acid regurgitation		Meal-related distress subscale (item 25–27)
		12	Sucking sensations in the epigastrium		Indigestion subscale (item 14–17)
		13	Nausea and vomiting		Diarrhea subscale (item 19, 20, 22)
		14	Borborygmus		Constipation subscale (item 18, 21, 23)
		15	Abdominal distension		Dumping subscale (item 30, 31, 33)
		16	Eructation		
		17	Increased flatus		Total symptom scale (above seven subscales)
		18	Decreased passage of stools		
		19	Increased passage of stools		
		20	Loose stools		
		21	Hard stools		
		22	Urgent need for defecation		
		23	Feeling of incomplete evacuation		
	Symptoms	24	Bile regurgitation		
		25	Sense of foods sticking		
		26	Postprandial fullness		
		27	Early satiation		
		28	Lower abdominal pains		
		29	Number and type of early dumping symptoms		
		30	Early dumping general symptoms		
		31	Early dumping abdominal symptoms		
		32	Early dumping abdominal symptoms		
		33	Late dumping symptoms		
Living status	Meals (amount) 1	34	Ingested amount of food per meal*		
		35	Ingested amount of food per day*		
		36	Frequency of main meals		
		37	Frequency of additional meals		
	Meals(quality)	38	Appetite*	Five-point Likert scale	Quality of ingestion subscale* (item 38–40)
		39	Hunger feeling*		
		40	Satiety feeling*		
	Meals (amount) 2	41	Necessity for additional meals		
	Social activity	42	Ability for working		
QOL	Dissatisfaction (QOL)	43	Dissatisfaction with symptoms		Dissatisfaction for daily life subscale (item 43–45)
		44	Dissatisfaction at the meal		
		45	Dissatisfaction at working		

In items or subscales with*; higher score indicating better condition. In items or subscales without*; higher score indicating worse condition. Each subscale is calculated as the mean of composed items or subscales except PCS or MCS of SF-8. Item 29 and 32 do not have score. Then, they were analyzed separately

Additionally, 12 items associated with intake of food, working, and satisfaction degree for daily life were assessed in this study. Food ingestion contains five regarding the ingested amount of oral intake (from 34 to 37, 41) and three pertaining the property of ingestion (from 38 to 40). Another associated with working (42), and the remaining three pertain the satisfaction degree for everyday life (from 43 to 45).

The twenty-three symptom items consist of a seven-grade Likert scale. All other excluding 1, 4, 29, 32, and 34–37 consist of a five-grade Likert scale. Higher scores point out better situations in 1–8, 34, 35 and 38–40. Conversely, higher scores point out worse situations in 9–28, 30, 31, 33, and 41–45. The primary result scale was polished by reinforcement and excerption. Twenty-three items of symptom were merged into seven subscales (SS) of symptom by analyzing factors [6], as shown in Table 1. Evaluation contains score of total symptoms, quality of ingestion SS, dissatisfaction for daily life SS, physical component summary (PCS), and mental component summary (MCS) in the SF-8 as primary result scale. Furthermore, we picked up the data for primary result scale: weight change, quantity of food intake, requirement of additional food, ability to working, discontent about conditions, discontent about food, and discontent about working. Individual SS points signify average of draw up items, and average of seven symptom SS signifies the entire symptom points (Table 1).

### Methods of study

We used a central registration system to register consecutive patients in this study. The questionnaire was delivered to all patients who are eligible when they visited to involved institutions. It is ordered for patients to turn back the format to the data center by mail. QOL data based on questionnaires were adapted to each enrolled data composed from case report forms. We registered this study in UMIN-CTR (No. 000002116). Approval of the Ethics Review Board was obtained in all institutions to participate PGSAS and submit data. Informed consent in writing was held from all enrolled cases.

### Statistical analysis of data

To compare among the groups, the analysis of variance (ANOVA) and Fisher's exact test were used. In case the *P* value was <0.05 in Fisher's test, residual analysis was added. In case the *P* value of ANOVA was less than 0.1, Tukey was conducted. When the *P* values were <0.1 in Tukey, Cohen’s *d* was performed for the purpose of effect size. *P* < 0.05 was considered statistically significant. Cohen’s *d* means the effect of the variable of individual cause: the effect size from 0.2 to 0.5 indicates a small difference clinically; from 0.5 to 0.8 indicates a moderate effect; and ≥0.8 denotes a large effect clinically. Data analysis was conducted making use of JMP12.0.1 (SAS Institute Inc.).

## Results

### Retrieving the questionnaire

**Fig. 1:**
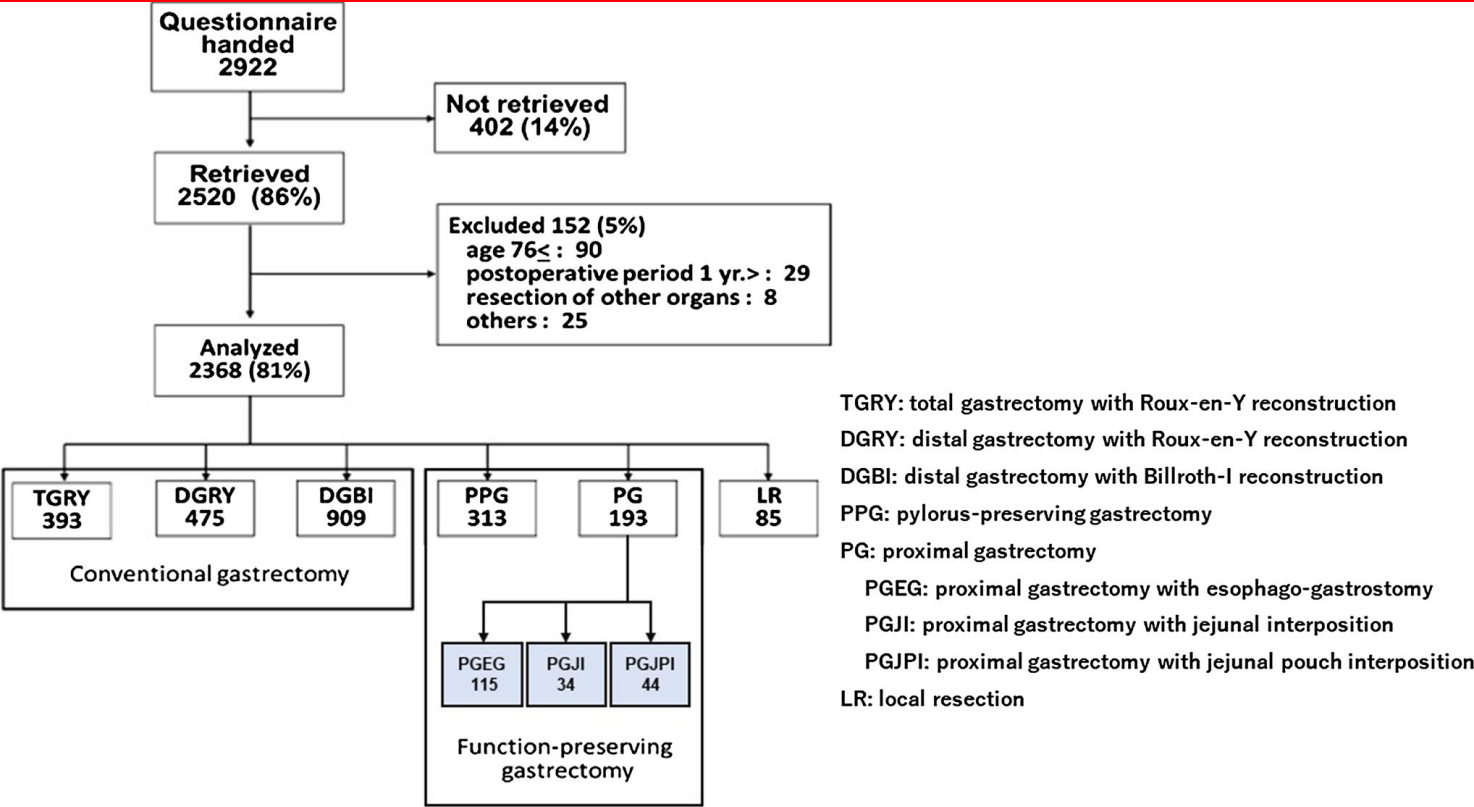
Outline of this study

A total of 2,520 (86.2%) questionnaires were screened, and 152 were thought to be not eligible for age over 75 years (*n* = 90), postoperative period within one year (*n* = 29), combined surgical removal (*n* = 8), and other causes (*n* = 25). Finally, 2,368 questionnaires (81%) were determined to be eligible. PG was 193 cases in all 2,368, and among them, 115 cases were performed by PGEG, 34 cases by PGJI, and 44 cases by PGJPI (Fig. 1). Patient reported outcomes of these 193 cases were picked up for analyses.

### Characteristics of patient

Characteristics of the 193 patients are shown in Table 2. There were no significant differences among patients receiving the three reconstruction methods regarding the background such as age, gender, postoperative period, approach, and preservation of the vagal celiac branch. However, about the size of remnant stomach, proportion of patients with the remnant stomach size greater than or equal to 2/3 of the whole stomach was significantly larger in the PGEG (86.6%) and significantly smaller in the PGJPI (14.3%). In contrast, patients with the size of remnant stomach that amounted to around 1/2 of the whole stomach were significantly more prevalent in the PGJPI (82.8%) and PGJI (59.4%) when compared with the PGEG (13.4%).

**Table 2:** Patients characteristics

Reconstruction method	PGEG		PGJI		PGJPI		*P*-value
Number	115		34		44		
Age (yr)^a^	64.1 ± 7.6		64.6 ± 7.3		61.8 ± 8.0		0.190a
Sex: Male/Female (N)	88/27		22/12		29/14		0.285b
Postoperative period (mo)^a^	37.8 ± 26.1		45.0 ± 31.1		43.9 ± 29.7		0.279a
Approach: Laparoscopic/Open (N)	17/98		8/26		8/35		0.475b
Celiac branch: preserved/not preserved (N)	49/64		11/23		23/18		0.115b
Size of the remnant stomach: N (%)							
Greater than or equal to 2/3	97	(86.6%)	13	(40.6%)	5	(14.3%)	< 0.001b
	*P* = 0.003c		*P* = 0.096c		*P* < 0.001c		
Around 1/2	15	(13.4%)	19	(59.4%)	29	(82.8%)	
	*P* < 0.001c		*P* = 0.021c		*P* < 0.001c		
Less than or equal to 1/3	0	(0%)	0	(0%)	1	(2.9%)	
	*P* = 0.429c		*P* = 0.672c		*P* = 0.069c		

^a^Mean ± SD

a: ANOVA, b: Chi-square test, c: residual analysis

### Assessments of QOL

The analysis of the 19 primary result scale of PGSAS-45 was performed using ANOVA and Tukey (Table 3). The quality of ingestion SS was better in the PGJI significantly compared with the PGEG (*P* = 0.022, Cohen’s *d* = 0.57) and PGJPI (*P* = 0.050, Cohen’s *d* = 0.59) (Table 3). The PGJPI showed better compared to the PGEG in several main outcome measures including food-related distress SS (*P* = 0.062, Cohen’s *d* = 0.39), constipation SS (*P* = 0.052, Cohen’s *d* = 0.42), dumping SS (*P* = 0.076, Cohen’s *d* = 0.40), dissatisfaction at working (*P* = 0.050, Cohen’s *d* = 0.42), and dissatisfaction for dairy life SS (*P* = 0.047, Cohen’s *d* = 0.43) with marginal meaning (Table 3).

**Table 3:** Multiple comparison of postoperative QOL among PGEG, PGJI, and PGJPI

	PGEG	*n* = 115	PGJI	*n* = 34	PGJPI	*n* = 44	ANOVA	Tukey		
	Mean	SD	Mean	SD	Mean	SD	*P* value		*P* value	Cohen's *d*
Esophageal reflux SS	2.0	1.0	2.1	1.0	2.0	0.9	0.895			
Abdominal pain SS	1.7	0.8	1.6	0.6	1.7	0.6	0.732			
Meal-related distress SS	2.8	1.2	2.6	0.9	**2.3**	1.0	0.075	PGEG versus. PGJPI	0.062	0.39
Indigestion SS	2.1	0.7	2.2	0.9	2.2	1.0	0.879			
Diarrhea SS	2.0	1.1	1.9	0.9	1.8	0.8	0.372			
Constipation SS	2.4	1.1	2.4	1.1	**2.0**	1.0	0.061	PGEG versus. PGJPI	0.052	0.42
Dumping SS	2.2	1.1	1.9	0.7	**1.8**	0.9	0.053	PGEG versus. PGJPI	0.076	0.40
Total symptom score	2.1	0.7	2.1	0.6	1.9	0.7	0.267			
Change in BW*	-0.1	0.1	-0.1	0.1	-0.1	0.1	0.424			
Ingested amount of food per meal*	6.4	2.0	6.8	1.8	6.5	1.8	0.489			
Necessity for additional meals	2.0	0.8	2.2	0.8	1.9	0.8	0.295			
Quality of ingestion SS*	3.5	1.0	**4.0**	0.8	3.5	1.0	0.022	PGJI versus. PGJPI	0.050	0.59
								PGJI versus. PGEG	0.022	0.57
Ability for working	2.1	0.9	1.8	0.8	1.8	0.9	0.221			
Dissatisfaction with symptoms	2.1	1.0	2.0	0.9	1.8	0.7	0.169			
Dissatisfaction at the meal	2.8	1.1	2.7	1.1	2.5	1.1	0.259			
Dissatisfaction at working	2.2	1.1	2.1	1.0	**1.7**	1.0	0.060	PGEG vs. PGJPI	0.050	0.42
Dissatisfaction for daily life SS	2.3	0.9	2.2	0.8	**2.0**	0.8	0.060	PGEG vs. PGJPI	0.047	0.43
PCS of SF-8*	49.3	6.3	50.5	5.2	49.4	6.3	0.610			
MCS of SF-8*	48.9	5.8	49.0	5.3	49.4	6.9	0.895			

## Discussion

The Japanese gastric cancer treatment guidelines version 4 proposed PG as selection for cT1cN0 adenocarcinoma existing on one-thirds of upper part of the stomach where over half of the distal stomach can be preserved [7], and PG has long been covered by the health insurance in Japan. Therefore, function-preserving PG is increasingly applied for them in Japan with the expectation of better QOL by preserving the both of secretion and motor activity of the remnant stomach. Additionally, importance of PG will increase more and more in the future with raising incidence of gastric cancer in early stage existing on one-thirds of upper part of the stomach. However, no prevailing consent exists regarding the optimal reconstructive method in PG with large-scale clinical trials at present [4, 18]. It has been discussed for a long time whether PG was in any ways superior to TG as an operative procedure for early stage cancer existing on one-thirds of upper part of the stomach [3, 4]. However, in a multicenter study focused on the analyses of self-entry-type questionnaire, PGSAS-45, for gastric cancer patients in early stage, superiority of PG over TG in terms of postgastrectomy QOL was clearly proven [6]. Nevertheless, some articles indicated that PGEG is associated with high risk of reflux esophagitis, while PGJI and PGJPI may cause stagnation in addition to occasional difficulties in the endoscopic examination of the remnant stomach [19, 20], postulating that PG should not be recommended unconditionally. To encounter these arguments, various reconstruction methods have been proposed for PG, but the debate for the optimal method continues. In the present study, we compared the postgastrectomy QOL after PG between three frequently performed methods: PGEG, PGJI, and PGJPI using the aforementioned PGSAS data. Although there was no remarkable difference among the groups, the outcome after PGJPI was marginally better in various aspects including meal-related distress SS, constipation SS, dumping SS, dissatisfaction at working, and dissatisfaction for dairy life SS despite the fact that a greater proportion of patients had small remnant stomach.

The results of a previous PGSAS study that focused on patients who received PGEG revealed that the size of remnant stomach certainly affected postgastrectomy QOL after PG, and the larger remnant stomach was associated with superior QOL [21]. Another article also pointed out the size of the remnant stomach as an important factor [22]. In the current study, although the remnant stomach size was significantly larger in the PGEG group with the proportion of more than two-thirds being as large as 86.6%, patients who received PGJPI had superior results in several of the main outcome measures, including meal-related distress SS, constipation, dumping, dissatisfaction at working, and dissatisfaction for dairy life although the difference was marginal, possibly reflecting the small samples size of the PGJPI group. These results indicate that PGJPI is a candidate for the favorable reconstruction method that maintains better QOL, at least when the size of the remnant stomach is rather small as around a half of the whole stomach. Postgastrectomy syndrome appears strongly in proximal gastrectomy when the size of the remnant stomach is small, so quantity of diet is thought to be decreased. Whereas, in PGJPI, even if the real size of the remnant is small, retention ability that is equivalent to save a large remnant stomach is obtained by making substitute stomach. We consider this is one of the reasons why QOL of PGJPI was superior compared with PGEG in our study.

Recently, various new reconstruction methods or anastomotic procedures in PG such as double tract [12, 13], double-flap reconstruction, side overlap esophago-gastrostomy (SOFY) [23], and other original ingenuities of the surgeons are widely performed. We should continue to examine the usefulness of these new techniques until to determine the optimal reconstruction procedures in PG.

Limitations of this study include its retrospective design, wide variation in duration from surgery, and analysis with the limited number of cases. Despite efforts to analyze a large number of patients with PGSAS which eventually retrieved more than 2,000 questionnaires from 52 institutions, given the proportion of patients who undergo PG, only 193 could be used for the current analyses. Thus, the study was not sufficiently powered for some of the analyses. Additionally, the comparison between the three different reconstruction methods should have been biased by the fact that each surgeon or institution likely selected one’s favorite reconstruction method [24–26]. For example, we cannot deny the possibility that the reconstruction method was selected according to the remnant stomach size at the discretion of the surgeon, and that led to the significant difference in that parameter between the three reconstruction methods. However, at the present time, there is no study that compared the different reconstruction methods after PG with comparable number of cases with the current study.

## Conclusion

Although the differences in postoperative QOL among the three different reconstruction methods after PG were marginal, PGJPI was superior to PGEG in several main outcome measures of PGSAS-45 despite the fact that patients who underwent this mode of reconstruction had smaller remnant stomach. PGJPI could be a favorable reconstruction method after PG, especially when remnant size is relatively small.
